# Obesity, Insulin Resistance, and Colorectal Cancer: Could miRNA Dysregulation Play a Role?

**DOI:** 10.3390/ijms20122922

**Published:** 2019-06-14

**Authors:** Francesca Cirillo, Cecilia Catellani, Chiara Sartori, Pietro Lazzeroni, Sergio Amarri, Maria Elisabeth Street

**Affiliations:** Department of Mother and Child, 42123 Azienda USL–IRCCS di Reggio Emilia, Italy; francesca.cirillo@ausl.re.it (F.C.); cecilia.catellani@ausl.re.it (C.C.); chiara.sartori@ausl.re.it (C.S.); pietro.lazzeroni@ausl.re.it (P.L.); sergio.amarri@ausl.re.it (S.A.)

**Keywords:** obesity, insulin resistance, inflammation, microRNA, colorectal cancer, cancer

## Abstract

Obesity is associated with insulin resistance and low-grade inflammation. Insulin resistance is a risk factor for cancer. A recent chapter in epigenetics is represented by microRNAs (miRNAs), which post-transcriptionally regulate gene expression. Dysregulated miRNA profiles have been associated with diseases including obesity and cancer. Herein we report dysregulated miRNAs in obesity both in animal models and in humans, and we also document dysregulated miRNAs in colorectal cancer (CRC), as example of an obesity-related cancer. Some of the described miRNAs are found to be similarly dysregulated both in obesity, insulin resistance (IR), and CRC. Thus, we present miRNAs as a potential molecular link between obesity and CRC onset and development, giving a new perspective on the role of miRNAs in obesity-associated cancers.

## 1. Obesity, Chronic Inflammation, and Insulin Resistance

Obesity is a disease characterized by chronic low-grade inflammation and insulin resistance (IR). Adipose tissue acts as an endocrine organ and secretes a large number of proteins, which regulate metabolism, energy intake, and fat storage such as leptin, adiponectin, interleukin- (IL-) 6, and tumor necrosis factor alpha (TNF-α) [1]. The chronic inflammatory state created by high cytokine levels generates a pro-tumorigenic environment promoting angiogenesis [2]. In addition, proinflammatory cytokines, mainly delivered by macrophages, can induce IR [3].

IR is defined as “the inability of a known quantity of exogenous or endogenous insulin to increase glucose uptake and utilization in an individual as much as it does in a normal population” [4]. Another major complication of obesity is the risk of developing the metabolic syndrome defined as the increase in waist circumference, hypertension, glucose intolerance, and dyslipidemia. Both IR and metabolic syndrome have been shown to be conditions predisposing tumoral development.

The link between metabolic alterations and tumors is mainly represented by changes in insulin, inflammation, and insulin-like growth factor (IGF) system [2,3,5].

## 2. Obesity and Cancer

Obesity is associated with the development of several cancers. Indeed, at least 12 types of cancer related to obesity have been described in the literature: colorectal, esophageal, gallbladder, gastric cardia, kidney, liver, intrahepatic bile duct, pancreatic, thyroid, uterine corpus, breast and ovarian cancers, and multiple myeloma [6] have been described and an increased risk has been reported.

Different neoplastic forms have different ages of onset, however an early increase in body weight has been shown to be related with the age of onset of some tumors. An anticipated age of diagnosis has been observed for colorectal, endometrial, pancreatic cancers, and multiple myeloma in patients who developed obesity in childhood [7].

As mentioned above, obesity is characterized by a status of chronic low-grade inflammation, which is also associated with several types of cancer. The link between inflammation and cancer is mainly represented by oxidative stress and cytokine and adipokine release [2].

Leptin is produced by adipose tissue and its levels are increased in obesity. Leptin acts on the hypothalamus mediating food intake and energy homeostasis. This hormone also stimulates cell growth, migration, and production of cytokines by macrophages. The promoting action of leptin on tumor development is supposed to be mediated by inducing the activation of proangiogenic factors [3].

Adiponectin, which is also produced by adipocytes and involved in energy homeostasis, is negatively correlated with BMI and inflammatory cytokine levels, and inhibits angiogenesis and inflammation. In CRC, its level has been shown to be inversely correlated with the risk and stage of cancer [3].

CRC can be considered an example of an obesity-related tumor as many studies have shown how environmental factors, such as weight gain, diet, level of physical activity, IR, and smoking increase its incidence [8].

Metabolic alterations also play a role in the development of CRC. Kim et al. observed an increased risk of CRC in obese men with metabolic syndrome in contrast with those obese but without metabolic alterations [9]. 

## 3. Insulin Resistance (IR) and Cancer 

Abdominal obesity correlates with alterations in circulating insulin levels. IR and subsequent hyperinsulinemia and type 2 diabetes mellitus (T2D) are conditions with increased risk of cancer, especially CRC, cholangiocarcinoma, and cancers of the endometrium, pancreas, and liver; the latter is the most increased cancer in diabetic patients [10]. 

The link between IR, hyperinsulinemia, and cancer has been explained by changes in the expression of insulin receptors and IGF system peptides. There is a very strong association indeed among IGF-I, insulin receptors and insulin, IGF-I, and IGF-II [11]. The interaction between IGF-I and its receptor has an important antiapoptotic effect; likewise, insulin has the same action [12]. Furthermore, insulin, which is known to be a growth factor, binds with low affinity to the IGF-I receptor (IGF1R), stimulating cell proliferation. An IGF-I serum level within the upper part of the normal range has been reported to be associated with an increased risk of cancer in the general population [5]. 

Fetal isoforms of the insulin receptor have been described in tumor cells, binding both insulin and IGF-II with high affinity. Moreover, some cancer cells locally produce IGF-II, promoting tumor proliferation [13]. Interestingly, the expression of the fetal insulin receptor has been reported to be increased in CRC and liver cancer and would contribute with other genetic and environmental factors to the development of these neoplasias. Animal models with precancerous colon adenomas have an increased expression of insulin receptors and, in particular, of the fetal isoforms compared with the mature forms further supporting a role in the development of this cancer [13]. In addition, Lu et al. demonstrated that insulin triggered cell proliferation and could induce metastatic effects in human CRC [14].

In this review, we will highlight the role of microRNAs (miRNAs) as a potential link between obesity and cancer focusing on CRC due to its relationship with the early development of obesity and with alterations of the metabolic state, in particular with IR. 

## 4. Biology of MicroRNAs (miRNAs)

Epigenetics concerns heritable modulations of gene expression, which do not presuppose a variation in the DNA sequence but can persist among generations [15,16]. 

The most reported epigenetic modifications, among others, are DNA methylation, histone modifications, nucleosome repositioning/chromatin remodeling, and miRNAs.

MiRNAs are short non-coding RNAs that regulate gene expression at the post-transcriptional level [17]. They are becoming more and more attractive since changes underlie pathological conditions and explain the variability among phenotypes. Furthermore, they have been proposed as prognostic and diagnostic markers and some therapeutic strategies have been proposed to restore physiological miRNA levels [18].

They are often grouped in clusters within the human genome and can be localized both between gene sequences (inter-genic regions) or into intronic regions (intra-genic regions) and are usually transcribed as single polycistronic transcripts. This organization allows a concerted regulation of gene expression of about 30% of the entire mammalian genome [19].

### Biogenesis and Action of miRNAs

During miRNA biogenesis, RNA polymerase II transcribes miRNA genes and produces a primary transcript (pri-miRNA) [20], which is a long RNA containing miRNA sequences bended in hairpins.

At the nuclear level, DiGeorge syndrome critical region 8 (Dgcr8) protein binds the enzyme Drosha belonging to the RNAse III family and they cleave the pri-miRNA generating a shorter double stranded pre-miRNA [21]. 

The pre-miRNA, upon the translocation to the cytosol via XPO5:RAN·GTP complex, is bound by Dicer, a cytoplasmic RNAse III complexed with its cofactor TRBP [21].

In the cytosol level, the Dicer:TRBP converts the pre-miRNA in a miRNA duplex of about 21–24 nucleotides [22].

The RNA-induced silencing complex (RISC), loaded with the miRNA duplex, separates and selects the single stranded miRNA, which is about 22 nucleotide long and contains a seed region that recognizes the mRNA target usually at the 3′ untranslated regions (UTR) but also at the 5’UTR and into coding DNA sequence (CDS) regions [23].

As a result, miRNAs determine the repression of translation or degradation of target mRNAs [24]. A single miRNA can target several genes and a single gene can be targeted by different miRNAs, revealing a complex regulatory network [25,26]. 

MiRNAs can have different expression levels dependent on the tissue or organ studied, and this explains why the same miRNA can be described with changes in opposite directions within a same condition. 

To date, about 2300 human mature miRNAs have been identified, 1115 of which are included in miRBase [27]; most of them have putative targets predicted by means of in silico analysis and also have validated targets. About 8500 articles reported 4000 miRNAs and 23,000 target genes including a total 420,000 miRNA–target interactions, which are currently collected in miRTarBase [28].

Altered miRNA profiles have been related with pathological conditions as “disease signatures” [29]. 

## 5. MiRNAs in Obesity

Due to the role of miRNAs as post-transcriptional regulators, recent evidences relate miRNA dysregulation with altered adipogenesis, and IR in obesity [30,31]. Furthermore, miRNAs have also been reported to be involved in endothelial dysfunction and cardiovascular disease, which are frequent complications in obesity [32].

Obesity progression hinges on the coordinated interactions among adipocyte hypertrophy, hyperplasia, and angiogenesis. Hypertrophic and hyperplasic adipocytes are associated with increased fat mass in obesity [33]. This is proven by in vitro studies. In 3T3-L1 cells, the referring model of adipocytes for in vitro studies, the inhibition of miR-15a reduces adipocyte hypertrophy while it enhances hyperplasia [34] and miR-210 stimulates the formation of hypertrophic adipocytes and the accumulation of lipid droplets acting on the Wnt signaling pathway [35]. Furthermore, miR-448 overexpression reduces the expression of adipogenic genes and triglyceride accumulation [36]. 

In the following sections, the main findings concerning miRNAs dysregulated in animal models and in humans with obesity will be summarized, documenting the link between obesity and miRNA changes.

### 5.1. Changes in miRNAs in Animal Models of Obesity

In mice with obesity induced by a high-fat diet, miR-21 has been described to be overexpressed in epididymal fat and associated with an increased number of adipocytes and of signal transducer and activator of transcription 3 (STAT3), a key protein that responds to cytokines and growth factors [37], whereas if knocked-down, reduced weight and adipocyte size were observed [38]. Furthermore, it was found increased in plasma from obese and lean mice fed with a high-fructose diet [39].

MiR-24 was reported upregulated in liver in both diet-induced and genetic obese mice and targeted scavenger receptor class B member 1 (SRB1), a plasma membrane receptor for high density lipoprotein cholesterol (HDL-C); this was related with reduced HDL uptake, steroid hormone biosynthesis and lipid metabolism [40]. 

MiR-27a was described downregulated in mature adipocytes from obese mice with respect to lean mice, suppressing adipocyte differentiation. MiR-27a is also known to target peroxisome proliferator activated receptor gamma (PPARγ) [41], and interestingly, it is also upregulated in the liver of obese and lean mice on a high-fructose diet [39], in genetically obese mice, as well as in epididymal fat tissue [42].

MiR-30a was shown to improve insulin sensitivity and reduce inflammation in white adipose tissue from both obese mice and humans, putatively targeting signal transducer and activator of transcription 1 (STAT1) and counteracting interferon gamma (IFN-γ) action [43]. MiR-30b and miR-30c have been proven to promote beige fat development and regulate receptor-interacting protein 140 (RIP140) [44], of which deficiency determines a lean phenotype and resistance to obesity in mice [45]; furthermore, miR-30b was up-regulated in the liver of high-fat diet-induced obese rats and was positively correlated with hepatic steatosis while its inhibition improved insulin sensitivity [46]. 

MiR-103/107 cluster inactivation improved glucose tolerance and insulin sensitivity, while its gain of function impaired glucose tolerance in obese mice [47]. Further, miR-103 was found to be downregulated in adipocytes from obese mice [48].

MiR-143 was upregulated in the liver of both genetic and diet-induced obese mice and the up-regulation of miR-143 was related to impaired insulin sensitivity due to altered AKT serine/threonine kinase (AKT) regulation from oxysterol-binding-protein-related protein (ORP) 8, a target of miR-143. Consistently, its deficiency protected obese mice against IR [49]. Moreover, miR-143 was found up-regulated in the mesenteric fat pad from obese mice induced by high-fat diet and was related to changes in PPARγ [50], while it was downregulated in epididymal adipose tissue [48].

MiR-144-3p levels were increased in obese mice adipose tissue where targeted two corepressors of C/EBPα activity and associated with increasing adipogenesis [51]. 

MiR-146a-null mice fed with a high-fat diet have abnormal weight increase, hepatic complications, and altered glycemia. Therefore, miR-146a could play an anti-adipogenic role counteracting obesity development, hyperglycemia, and inflammation in visceral adipose tissue and liver [52]. 

MiR-205-5p, increased in obese mice, targets forkhead box O1 (FOXO1), a key transcription factor of insulin signaling [53]. 

MiR-328 levels have been described to be decreased in brown adipose tissue (BAT) in obese mice and to have a role in glucose and lipid homeostasis under chronic nutrient excess [54]. 

Mice deficient in miR-378-3p and miR-378a-5p became resistant to obesity due to high-fat diet and showed improved fatty acid metabolism targeting carnitine O-acetyltransferase, enhancing oxidative efficiency of insulin-sensitive tissues [55]. Consistently, miR-378-3p was found upregulated in adipose tissue from diet-induced obese mice [56].

In obese mice with renal dysfunction, knock down of miR-802, improved renal functional parameters and functions and reduced inflammation by targeting NF-κB-repressing factor [57].

Table 1 summarizes miRNAs dysregulated in conditions of obesity, insulin resistance in mouse and/or rat models of obesity, both at the systemic and tissue level. Some miRNAs show changes in opposite directions due to studies having been performed in different tissues or organs. 

### 5.2. Changes in miRNAs in Human Subjects with Obesity 

Many miRNAs have been studied in obese humans both at the systemic and cellular, tissue, and organ level and possible relationships with obesity and IR for single miRNAs are reported. 

#### 5.2.1. Dysregulation of Circulating miRNAs 

Several studies have investigated circulating miRNAs in obesity. Among the 18 differentially expressed miRNAs detected in blood using a global profiling approach from morbidly obese men with respect to lean subjects, miR-142-3p, miR-140-5p, and miR-222 were upregulated while miR-221, miR-15a, miR-520c-3p, miR-423-5p, and miR-130b, were downregulated. These results were also confirmed in a subset of morbidly obese patients after surgery-induced weight loss. [58]. Recently, seven miRNAs (miR-7-5p, let-7f-5p, miR-15b-5p, let-7i-5p, miR-320c, miR-205-5p, and miR-335-5p) were described to be differentially expressed in obese patients after bariatric surgery. Interestingly, these miRNAs targeted genes included in diabetes and IR-related pathways [59].

A global meta-analysis conducted systematically in PubMed on papers identifying circulating miRNAs useful for the diagnosis of obesity and T2D in humans, reported that miR-142-3p, miR-140-5p, and miR-222 were upregulated while miR-21-5p, miR-221-3p, miR-125-5p, and mir-103-5p were downregulated in obese patients, whereas miR-142-3p and miR-222 were commonly upregulated in obese and T2D patients. Furthermore, an in silico analysis of targeted genes and pathways suggested a potential role of these two latter miRNAs in metabolic features of both obese and T2D patients [60]. 

MiR-374a-5p was upregulated in metabolic healthy obese subjects and was related to a reduction in proinflammatory cytokines (CCL2/CCR2 axis), which contribute to IR [61]. 

A cross-sectional study reported that 15 circulating miRNAs (miR-221 and miR-28-3p, miR-125b, miR-328, miR-486, miR-142-3p, miR-130b, miR-423-5p, miR-532-5p, miR-140-5p, miR-16, miR-222, miR-363, miR-122, and miR-195) were dysregulated in prepubertal obese patients. Interestingly, two miRNAs were increased (miR-142-3p and miR-486), and two were decreased (miR-221 and miR-28-3p), which have been shown to be able to predict weight gain/loss in childhood during growth and could be associated with the risk of developing obesity-related complications in adulthood [62].

#### 5.2.2. miRNAs Dysregulated in Tissues

It is noteworthy that miRNAs showing changes in the circulation, considered as a signature of obesity, reflect to some extent those differentially expressed in cells, tissues, and organs in obese patients. 

MiR-17-5p and mir-132 expression levels were reported to be significantly different in omental fat and blood in obese patients with respect to lean individuals and their levels correlated with BMI, fasting blood glucose, and glycosylated hemoglobin [63]. 

MiR-20b was found to be down-regulated while miR-29a-3p and miR-29a-5p to be up-regulated in subcutaneous adipose tissue from morbidly obese individuals after weight loss intervention [64]. Furthermore miR-20b, miR-296, and Let-7f, which inhibit genes involved predominantly in the vascular endothelial growth factor (VEGF) and Wnt pathways, were differentially expressed in visceral adipose tissue of normoglycemic-obese patients with respect to T2D-obese subjects [65]. 

MiR-23a-3p and miR-181a-5p were downregulated in adipose tissue from obese and diabetic subjects and were negatively correlated with adiposity and IR [66].

The miR-27 family was described to be upregulated in omental multipotent stem cells isolated from patients with morbid obesity with respect to lean subjects, leading to a dysregulation of important pathways involved in early stages of adipogenic differentiation as the Wnt, TGFβ/Smad and PPARγ/ C/EBPα pathways [67].

Consistent with the findings in an obese mice model [48], miR-221 was upregulated in obese individuals and targeted adiponectin receptor 1 (ADIPOR1), which encodes for a receptor of adiponectin and ETS proto-oncogene 1 (ETS1), a member of ETS family of transcription factors, suggesting a relationship with the development of IR and T2D in obesity [68]. 

In a miRNA microarray analysis, miR-519d was found to be overexpressed in the subcutaneous adipose tissues of both obese men and women [69]. PPARα, a predicted target of miR-519d, was also demonstrated to be reduced in the same patients and, consistently, a suppression of miR-519d induced a reduction in the PPARα protein and promoted lipid accumulation in preadipocytes. The same effects on adipocyte differentiation were also shown for miR-143, suggesting a potential role for these two miRNAs in obesity. Furthermore, miR-143 and miR-652-3p were investigated in fat cells from obese IR women and, interestingly, both correlated positively with insulin-enhanced lipogenesis and white adipose tissue IR [70]. 

MiR-592 was reported to be downregulated in the liver of obese subjects and its overexpression in obese mice improved hepatic glucose metabolism directly targeting FOXO1. Moreover, miR-592 inhibition induced hyperglycemia, IR, and hepatic triglyceride accumulation in lean mice [71].

MiR-802 was described to be upregulated in the liver of obese human patients and to target hepatocyte nuclear factor 1-beta (Hnf1b), where reduction was related to impaired glucose tolerance and reduced insulin sensitivity [72].

A characterization of the exosome content, which represents a depot of miRNAs to be delivered in target tissues, detected 55 miRNAs differentially expressed in visceral adipocytes from obese young patients, which mainly targeted genes involved in the transforming growth factor beta 1 (TGF-β) and Wnt/β-catenin signaling pathways related to adipocyte differentiation [73]. In addition, a panel of 168 miRNAs was differentially expressed in adipocyte-derived exosomes from obese female patients after surgery compared with the levels they had before surgery and these miRNAs regulated gene targets mainly involved in insulin receptor signaling underlying the link between obesity and IR [74]. 

Table 2 resumes miRNAs dysregulated in conditions of obesity, insulin resistance in human subjects, both at the systemic and tissue level. 

## 6. MicroRNAs Linking Obesity to Colorectal Cancer (CRC)

Colorectal cancer (CRC) is one of the most frequent tumors diagnosed in both men and women and is the fourth cause of cancer-related death in the world [75]. Obesity has been found to be one of the major risk factors for the onset and development of CRC [76]. About 11% of CRCs have been associated with overweight and obesity in Europe. Indeed, epidemiological data ascribe a 30–70% increased risk of CRC to obesity associated with hyperinsulinemia and in particular to abdominal obesity [76,77,78]. Nevertheless, increased serum insulin levels and related IR have been reported as risk factors per se for the development of CRC [79].

Epidemiological studies reported that diet and lifestyle contribute to the increasing CRC incidence. Obesity increases the risk of CRC by 19%, whereas regular physical activity decreases this risk by 24% [80]. Furthermore, red meat excessive intake, moderate alcohol consumption, and smoking represents risk factors of CRC, while frequent intake of fruits, vegetables, whole-grain cereals, fish, milk, white meats, and soy derivatives are factors of primary prevention [80,81]. Strong evidences support that folic acid, calcium, and vitamin D decrease the CRC risk [82]. A possible mechanism on the involvement of body fat on CRC risk is based on obesity-related hormone levels, such as insulin, estrogens, and IGF-1, promoting carcinogenesis and counteracting apoptotic cell death [83].

The molecular mechanisms by which obesity contributes to CRC are still poorly understood. However, there are a few pathophysiological processes identified as potential triggers of CRC in obesity, mainly including IR as mentioned above and chronic low-grade inflammation, besides genetic factors, and an unhealthy diet [84]. Altered biochemical indexes including hyperinsulinemia [85], increased IGF-I bioavailability [86], increased pro-inflammatory cytokines [87], adipokines [88], and sex-steroid hormones [89] have all been identified as possible contributing factors to CRC. 

Recent data show that changes in circulating miRNA levels are found in several diseases, including CRC and obesity [90,91] and they are also influenced by lifestyle [92]. Furthermore, a possible role of miRNAs in CRC in obese subjects is yet poorly explored. 

Although associations of obesity with CRC [93,94,95,96] and of miRNAs with CRC [90,97,98,99,100,101,102,103,104,105,106,107,108,109,110,111,112] are well documented as well as evidences linking miRNAs to clinical features of obesity as IR [113,114,115,116] and inflammation [87,117] are well-founded, there is still a lack of data in the literature concerning the role of miRNAs as a potential link between obesity and CRC [118].

An interesting mechanism by which a dysregulation of a single miRNA in CRC contributes to the development and progression of CRC, through the amplification of the IGF-1R signaling, was identified in an in vitro study conducted in both human CRC cell lines and human CRC tissues. Specifically, the downregulation of miR-497 determined an upregulation of the IGF1R in CRC cells and the overexpression of miR-497 led to a reduction in endogenous IGF1R protein in CRC cells [119]. This contributed to CRC malignancy (inhibition of cell survival, proliferation, and invasion, and increased sensitivity to apoptosis induced by chemotherapeutic drugs). These effects were shown to be mainly mediated by inhibition of phosphatidylinositol 3-kinase/Akt signalling. Overall, these results suggested that the restoration of miR-497 levels could be a useful alternative approach to inhibit IGF-1R in CRC. Previously, this was shown also in a mouse model of CRC for miR-145 and miR-33a [120]. Moreover, miR-145 was described to be upregulated in the liver of both genetic and diet-induced obese mice [49], and miR-33a was decreased in white adipose tissue [39], and when knocked out, predisposed mice to obesity and IR [121]. These findings suggested that both miRNAs were related with obesity, IR, and cancer.

Interestingly, the above-mentioned miR-497, in a large-scale meta-analysis performed on human databases containing body mass index and waist-to-hip ratio (WHR) data, showed that the miR-497 gene was linked with WHR, showing that it was also involved in obesity [122]. Furthermore, high levels of miR-497 induced IR in the liver of rats fed with high-fat diet inhibiting insulin receptor gene expression [123]. Moreover, miR-497 was first shown to have a role in influencing CRC cell growth by targeting a downstream insulin signaling mediator, insulin receptor substrate 1 (IRS1), in a study conducted in human CRC tissues compared with the normal adjacent tissues. In vitro induced overexpression of miR-497 was found to be reduced in CRC tissues, inhibited proliferation, migration, and invasion capacity of CRC cells, and reduced phosphoinositide 3 kinase (PI3K)/AKT and mitogen-activated protein kinase/extracellular signal-regulated kinase (MAPK/ERK) signaling by targeting insulin receptor substrate 1 (IRS1), a downstream insulin signaling mediator. In conclusion, this study confirmed that miR-497 was able to inhibit the malignant features of CRC cells by targeting IRS1 [124]. 

Molecular mechanisms integrating mediators involved in metabolic pathways (insulin and leptin receptor signaling cascades) with carcinogenesis processes (CRC invasion and metastasis) have been described related also to other miRNAs. In human CRC-derived cell lines, insulin and/or leptin resistance, which usually occurs in obesity, interferes with the activation of mitogen-activated protein kinase 1/2 (MEK1/2) induced by insulin/leptin, which leads to an increase in miR-4443, in turn inhibiting nuclear receptor coactivator (NCOA)-1 and tumor necrosis factor receptor-associated factor (TRAF)-4, possibly causing tumor suppression and decreasing cell invasion [125]. 

Other miRNAs are likely to be regulated by the same cascade downstream of both insulin and leptin, contributing to the correlation between obesity/IR and cancer risk. Therefore, the following paragraph highlights the emerging role of miRNAs in the cross-communication between obesity and CRC. 

Few findings have described associations among obesity, CRC, and miRNAs, and some are from a randomized study conducted in mice after diet-induced obesity or caloric restriction. Diet-induced obesity increased the number of colon tumors, inflammatory cytokines, IGF-I, and proliferation [126]. Opposite results were obtained in caloric-restricted mice. In particular, nine miRNAs (miR-425, miR-196, miR-155, miR-150, miR-351, miR-16, let-7, miR-34, and miR-138) were found to be differentially expressed in both diet-induced obesity and caloric-restricted mice compared with controls [126]. Interestingly, most of these miRNAs were also described to be dysregulated in simple obesity. The overexpression of miR-425 in mice was regulated by PPARγ and was also capable of inhibiting mitogen-activated protein kinase 14 (Mapk14), a negative regulator of adipogenesis [127]. PPARγ dysregulation was reported to increase the risk of CRC in obese subjects as previously mentioned [128]. 

Changes in miR-155 in obese mice can induce obesity and non-alcoholic fatty liver disease (NAFLD) and led to an increase in resistin, which regulates insulin sensitivity [129]. 

MiR-150 KO mice fed with a high-fat diet showed reduced body weight and increased mechanistic target of rapamycin kinase (mTOR) expression, which enhanced leptin levels [130]. Leptin is altered in obesity and related to CRC [3]. 

Furthermore, as previously described, miR-16 was dysregulated in prepubertal obese patients in a cross-sectional study that analyzed circulating miRNAs [62]. The Let-7 family has been shown to act as a pro-adipogenic factor targeting high-mobility group AT-Hook 2 (HMGA2) protein, which reduces fat mass in obese leptin-deficient mice [131,132] suggesting, once again, a role for leptin and obesity in CRC [3]. MiR-34 was described to be increased also in humans with NAFLD and with T2D [133,134] and few experimental evidences indicate that increased miR-34a levels in the liver are associated with metabolic alterations [135]. T2D further increases the risk of cancer in obese subjects [10]. 

MiR-138 and miR-376a are specific of obese patients, and miR-138 combined with miR-503 differentiated diabetic from obese diabetic patients [91].

Further information relative to an interplay between metabolic disorders and CRC mediated by miRNAs was given by an animal and an in vitro study that evaluated the effects of hypercholesterolemia on the incidence and severity of CRC [136]. Hypocholesterolemia promoted the up-regulation of miR-101c, which, in turn, reduced ten eleven translocation (Tet)-1 in hematopoietic stem cells (HSC), causing a decrease in the expression of several genes involved in natural killer T- and γδ T-cell differentiation in the thymus, and colon submucosa at the first stages of carcinogenesis. These findings suggested a new epigenetic mechanism by which a comorbidity of obesity (hypercholesterolemia) reduced lineage priming of HSC toward immune cells, thereby eluding normal immunosurveillance against tumors.

Recently, miR-21, which was associated with obesity and T2D in mice as previously mentioned [38], was shown to have an oncogenic role in CRC since it was found significantly upregulated in CRC at variance with colorectal adenoma and non-neoplastic mucosa [137].

A milestone regarding the investigation on the role of circulating miRNAs in CRC associated with obesity is represented by a study conducted in CRC patients with and without obesity. Three circulating miRNAs (miR-27b, miR-130b, and miR-138) were found to be increased and to correlate negatively with peroxisome proliferator-activated receptor gamma (PPARγ), evaluated in peripheral blood mononuclear cells (PBMC). PPARγ is known to exhibit tumor suppressor effects [128], and the increase in these miRNAs was associated with the CRC risk in the obese subjects. According to these findings in CRC, miR-27b-3p overexpression enhanced visceral lipid accumulation and inhibited browning in both white adipose tissue [138] and epididymal fat tissue [42] in obese mice. Furthermore, as previously reported (see Section 5.2.1), miR-130b was downregulated in serum of morbidly obese with respect to lean subjects and in patients after surgery-induced weight loss [58] as well as prepubertal obese patients [62].

A recent review documented the ability of adipose tissue to induce a dysregulation in miRNA levels with oncogenic and tumor suppressor effects [139]. Indeed, adipokines, secreted by adipose tissue and with hormone-like functions [140], increase in obesity and exert a regulatory function on miRNA levels [141]. 

## 7. Conclusions

**Figure 1:**
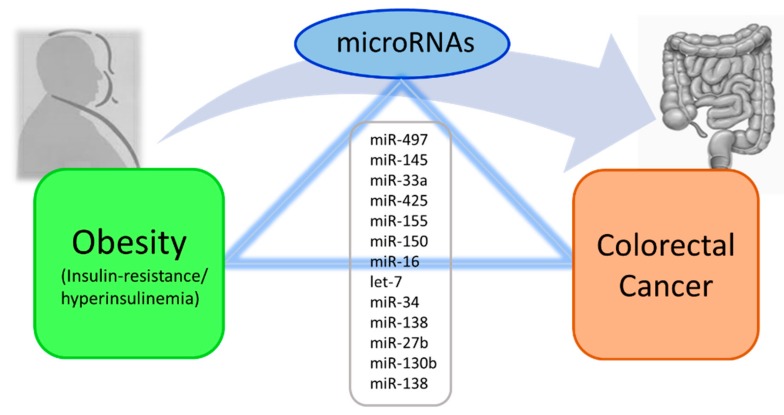
Dysregulated microRNAs in common between obesity and colorectal cancer (CRC), possibly linking the two conditions.

This review highlights changes in miRNAs involved with insulin sensitivity, glucose tolerance, and lipid metabolism both in obesity and CRC. All the metabolic changes have been shown to be related with the risk of CRC in obesity, and some of the described miRNAs were found to be similarly dysregulated in obesity, IR, and CRC (Figure 1). Therefore, miRNAs could represent a potential molecular link between the metabolic alterations related with obesity and CRC onset and development. Overall, these findings shed new light on obesity as a CRC risk factor where miRNA dysregulation potentially plays a role. 

## Figures and Tables

**Table 1:** MiRNAs dysregulated in animal models of obesity.

miRNA	Up/Down Regulation	Condition	Ref.
miR-21	↑	Adipocyte hyperplasia in epididymal fat	[37,39]
	↓	Obesity and T2D	[38]
miR-24	↑	Obesity (in liver)	[40]
miR-27a	↓	Obesity in adipocytes	[41]
	↑	Obesity (in liver and epididymal fat tissue)	[39,42]
miR-30a	?	Improves insulin sensitivity and reduces inflammation in obesity	[43]
miR-30b	↑	Obesity (in liver); promotion of beige fat development	[46,46]
miR-30c	?	Promotion of beige fat development in obesity	[44]
miR-103/107 cluster	↓	Improvement of glucose tolerance and insulin sensitivity in obesity	[47,48]
miR-143	↑	Impairment of insulin sensitivity in the liver, its deficiency protects mice from IR; obesity (in mesenteric fat pads)	[49,50]
	↓	Obesity (in epididymal adipose tissue)	[48]
miR-144-3p	↑	Obesity (in adipose tissue)	[51]
miR-146a	KO	Abnormal weight increase, hyperglycemia, and inflammation in obesity	[52]
miR-205-5p	↑	Obesity: targeting FOXO1	[53]
miR-328	↓	Obesity (in BAT), involved in glucose homeostasis	[54]
miR-378-3p	KO	Resistance to obesity under high-fat diet, improvement of fatty acid metabolism and oxidative efficiency in insulin-sensitive tissues	[55]
	↑	Obesity	[56]
miR-378a-5p	KO	Obesity resistance under high-fat diet, improvement of fatty acid metabolism and oxidative efficiency in insulin-sensitive tissues	[55]
miR-802	KO	Improvement of renal function in obesity, reduction of inflammation	[57]

Abbreviations: ↑ = up-regulated; ↓ = down-regulated; ? = unknown; miRNA = microRNA; T2D = type 2 diabetes mellitus; KO = knock-out; FOXO1 = forkhead box O1; BAT = brown adipose tissue.

**Table 2:** MiRNAs dysregulated in human obese subjects.

miRNA	Up/Down Regulation	Condition	Ref.
Let-7f	↑/↓	Obesity and T2D	[65]
let-7f-5p	↑/↓	Obesity: changes after bariatric surgery	[59]
let-7-i-5p	↑/↓	Obesity: changes after bariatric surgery	[59]
miR-7-5p	↑/↓	Obesity: changes after bariatric surgery	[59]
miR-15a	↓	Obesity	[58]
miR-15b-5p	↑/↓	Obesity: changes after bariatric surgery	[59]
miR-16	↑/↓	Obesity	[62]
miR-17-5p	↑/↓	Associated with BMI, FBG, HbA1c in obesity	[63]
miR-20b	↑	Obesity and T2D	[64,65]
miR-21-5p	↓	Obesity	[60]
miR-23a-3p	↓	Obesity and T2D (negatively correlated with adiposity and IR index)	[66]
miR-27	↑	Obesity	[67]
miR-28-3p	↓	Obesity	[62]
miR-29a-3p	↓	Obesity	[64]
miR-29a-5p	↓	Obesity	[64]
miR-103-5p	↓	Obesity	[60]
miR-122	↑/↓	Obesity	[62]
miR-125-5p	↓	Obesity	[60]
miR-125b	↑/↓	Obesity	[62]
miR-130b	↑/↓	Obesity	[58,62]
miR-132	↑/↓	Associated with BMI, FBG, HbA1c in obesity	[63]
miR-140-5p	↑/↓	Obesity	[58,60,62]
miR-142-3p	↑	Obesity and T2D	[58,60,62]
miR-143	?	Promotion of lipid accumulation, positively associated with insulin-enhanced lipogenesis and WAT IR in obesity	[70]
miR-181a-5p	↓	Obesity and T2D (negatively correlated with adiposity and IR index)	[66]
miR-195	↑/↓	Obesity	[62]
miR-205-5p	↑/↓	Obesity: responsive to bariatric surgery	[59]
miR-221	↑/↓	Obesity and T2D	[58,62,68]
miR-221-3p	↓	Obesity	[60]
miR-222	↑/↓	Obesity and T2D	[58,60,62]
miR-296	↑/↓	Obesity and T2D	[65]
miR-320c	↑/↓	Obesity: responsive to bariatric surgery	[59]
miR-328	↑/↓	Obesity	[62]
miR-335-5p	↑/↓	Obesity: responsive to bariatric surgery	[59]
miR-363	↑/↓	Obesity	[62]
miR-374a-5p	↑	Metabolic syndrome	[61]
miR-423-5p	↑/↓	Obesity	[58,62]
miR-486	↑	Obesity	[62]
miR-519d	↑	Obesity: promotes lipid accumulation	[69]
miR-520c-3p	↓	Obesity	[58]
miR-532-5p	↑/↓	Obesity	[62]
miR-592	↓	Obesity (in liver)	[71]
miR-652-3p	?	Positively associated with insulin-enhanced lipogenesis and WAT IR in obesity	[70]
miR-802	↑	Obesity impaired glucose tolerance and reduced insulin sensitivity in liver	[72]

Abbreviations: ↑ = up-regulated; ↓ = down-regulated; ? = unknown; miRNA = microRNA; T2D = type 2 diabetes; KO = knock-out; FOXO1 = forkhead box O1; BAT = brown adipose tissue; BMI = body mass index; FBG = fasting blood glucose; HbA1c = glycated haemoglobin; WAT = white adipose tissue; IR = insulin resistance.

## Abbreviations

ADIPOR1	Adiponectin Receptor 1
AKT	AKT Serine/Threonine Kinase
BMI	Body mass index
BAT	Brown adipose tissue
CCL2	C-C Motif Chemokine Ligand 2
CCR2	C-C Motif Chemokine Receptor 2
CDS	Coding DNA sequence
CRC	Colorectal cancer
Dgcr8	DiGeorge Syndrome Critical Region Gene 8
ETS1	ETS Proto-Oncogene 1
FOXO1	Forkhead Box O1
HSC	Hematopoietic stem cells
HDL-C	High density lipoprotein-Cholesterol
HMGA2	High Mobility Group AT-Hook 2
Hnfb1	HNF1 Homeobox B
IGF1R	IGF-I receptor
IRS1	Insulin Receptor Substrate 1
IGF	Insulin-like growth factor
IR	Insulin resistance
IFN	Interferon
IL	Interleukin
KO	Knock-out
mTOR	Mechanistic Target Of Rapamycin Kinase
miRNA	microRNA
MAPK	Mitogen-Activated Protein Kinase
MEK 1/2	Mitogen-Activated Protein Kinase Kinase 1/2
NAFLD	non-alcoholic fatty liver disease
NF-κB	Nuclear Factor Kappa B
NCOA-1	Nuclear Receptor Coactivator-1
ORP8	Oxysterol-binding protein-related protein 8
PBMC	Peripheral blood mononuclear cells
PPAR	Peroxisome Proliferator Activated Receptor
PI3K	Phosphoinositide-3-Kinase
pri-miRNA	Primary miRNA
RIP140	Receptor-Interacting Protein 140
RISC	RNA-induced silencing complex
SRB1	Scavenger Receptor Class B Member 1
STAT	Signal Transducer And Activator of Transcription
SHH	Sonic Hedgehog Signalling Molecule
Tet-1	Tet Methylcytosine Dioxygenase 1
TRBP	Trans-Activation Responsive RNA-Binding Protein
TGF-β	Transforming Growth Factor Beta 1
TRAF-4	Tumor Necrosis Factor receptor associated factor (TRAF)-4
T2D	Type 2 diabetes mellitus
UTR	Untranslated region
VEGF	Vascular Endothelial Growth Factor
WHR	Waist-to-hip ratio
